# Area-Specific Assessment of Stratum Corneum Hydration and Transepidermal Water Loss in Pediatric Patients With Atopic Dermatitis

**DOI:** 10.1155/drp/2376970

**Published:** 2025-01-13

**Authors:** Bo Yeon Kwon, Dohyeong Kim, Kyungmin Shim, Cindy Nguyen, Hee Chul Lee, Daeshik Kang, Hohyun Kim, SungChul Seo

**Affiliations:** ^1^The Institute for Environmental Health and Safety, Seokyeong University, Seoul, Republic of Korea; ^2^School of Economic, Political and Policy Sciences, University of Texas at Dallas, Richardson, Texas, USA; ^3^The Samsung Kids Pediatric Adolescent Clinic Center, Seoul, Gyeonggi-do, Republic of Korea; ^4^Department of Mechanical Engineering, Ajou University, Suwon, Gyeonggi-do, Republic of Korea; ^5^Department of Nano, Chemical and Biological Engineering, Seokyeong University, Seoul, Republic of Korea

## Abstract

SCORring atopic dermatitis (SCORAD) is widely used to assess the severity of atopic eczema, but score systems based on the entire body may be limited in effective monitoring and intervention. It is crucial to monitor moisture levels in each affected body part, but empirical research is still lacking. The objective of this study was to analyze the levels of stratum corneum hydration (SCH) and transepidermal water loss (TEWL) in atopic dermatitis (AD) patients, focusing on the presence and location of atopic lesions at different body sites. The levels of TEWL and SCH were measured using the AF200 AquaFlux and the Corneometer, respectively, at 15 body sites. 98 children under the age of 10 were measured, including 83 AD patients and 15 in the control group. Patients were also assessed with SCORAD and for the presence of atopic lesions at each body site. 58.7% of AD patients had lesions in the antecubital fossa and popliteal fossa, with corresponding low SCH levels and high TEWL in the upper body. The differences in TEWL between the control group and AD patients were confirmed significant in the neck and antecubital fossa regions, while differences in SCH were identified in the face, antecubital fossa, and popliteal fossa regions. A higher TEWL was found among AD patients with atopic lesions in the face and ankle. This study suggests that continuous monitoring of SCH and TEWL levels at specific body sites can provide insights into identifying vulnerable body areas to AD and supplement the SCORAD system for more effective clinical intervention and prevention strategies.

## 1. Introduction

Atopic dermatitis (AD) is increasingly recognized as the first symptom in the progression of allergic diseases, marking the beginning of the “allergic march,” as highlighted in recent studies [1, 2]. Several previous studies have confirmed that children with AD are more likely to develop other allergies such as food allergies, allergic rhinitis, and asthma as they grow up, affecting their quality of life [3–5]. In addition, AD has several visual characteristics that can be identified as early as infancy, enabling early diagnosis. If detected at this stage, appropriate treatment and prevention efforts can be implemented, serving as an intervention against the progression of the “allergic march.” Therefore, early diagnosis and intervention are crucial in managing AD, as they improve long-term outcomes and help prevent the development of more severe allergic conditions [6–8]. The exact causes of AD have not been fully identified, but several complex factors such as genetics [9], environmental factors [10, 11], and immune system abnormalities [12] are known to contribute to the disease's pathophysiology. Recent studies have demonstrated that transepidermal water loss (TEWL) not only leads to skin barrier disruption and chronic inflammation but also plays a critical role in the onset and exacerbation of AD [13, 14]. In particular, the decrease in stratum corneum hydration (SCH) [14, 15] and the increase in TEWL [16, 17] are known to be closely linked to the exacerbation and onset of AD. Consequently, replenishing the skin barrier through skin moisturization is considered one of the most important factors in the treatment and management of AD [18].

To implement targeted proactive moisturization management as a preventative measure, it is essential to continuously monitor the skin moisture levels in the affected areas. However, current methods used in hospitals to evaluate AD severity focus primarily on symptoms, rather than on skin moisture levels. Several composite severity indices have been developed to assess the severity of atopic dermatitis, such as SCORAD, the Severity Scoring of Atopic Dermatitis, the Children's Dermatology Life Quality Index (CDLQI), and the Nottingham Eczema Severity Score [19]. Each evaluation method listed contains measured variables that lack necessary objectivity [20, 21] and lack standardization in terms of which body parts should be measured alongside how these measures and symptoms from multiple sites should be combined. Then, it varies further based on the patient's clinical symptoms and medical history [23]. This lack of standardization and objectivity results in suboptimal care. The SCORAD index, which has been widely used as a diagnostic tool for AD, is known for its appropriate validity, responsiveness, internal consistency, and observer reliability [20, 21], but also criticized for insufficient interobserver reliability [22] and unclear interpretability and feasibility [23]. The calculation of the index involves assessing the entire body, encompassing the overall severity of symptoms in a single numerical value, rather than using targeted data from selectively affected areas. The “Objective SCORAD” diagnostic method was developed to improve the objectivity of SCORAD; however, it still relies on evaluating the entire body [24, 25].

Previous studies attempted to assess whether SCORAD is correlated with skin pH level, moisture content, and moisture loss in patients with AD, resulting in a severe lack of data regarding on site-specific measurements [19, 23]. They have acknowledged the limitations of SCORAD and have attempted to analyze AD by observing specific affected body sites instead of using a full body calculation. A study by Choi et al. [23] discovered that there were differences in the measurement of TEWL and capacitance between extrinsic AD and intrinsic AD depending on the body site that was measured. Their data demonstrated that there was significant variance depending on the body site that was measured, such as the antecubital fossa having “higher TEWL and decreased capacitance” in both morphologies of AD, whereas intrinsic AD had “no significant differences of TEWL and hydration in the forehead, cheek, and the back of leg” [23]. It was argued that determining these key differences in symptom presentation per patient is crucial in treatment. A follow-up study by Hon et al. [19] determined that the antecubital flexure and the mid-forearm were convenient places to measure skin hydration and TEWL. This study also determined that there were correlations between acute and chronic scores of AD disease severity and quality of life based on their measured areas. While these studies were considered revolutionary during their time, they also had severe limitations, primarily in their small patient populations and methodology used to collect their data. With improved technology and data collection methods, their studies can be significantly expanded upon.

Skin moisture, which directly correlates with the symptoms of AD, has been identified as a biomarker, as moisture content and loss in the skin of AD patients are related to symptom severity [19]. However, since current diagnostic and analysis methods lack the capability to monitor specific body sites, complementary methods and further research are necessary to enable continuous site-specific monitoring. This study aims to construct a database of moisture content in different areas of patients' skin to analyze its correlation with symptoms, thereby identifying priority sites for continuous monitoring. This approach seeks to enhance therapeutic care and clinical outcomes by enabling more objective diagnosis and reducing reliance on patients' subjective symptom assessments.

## 2. Materials and Methods

### 2.1. Materials

This study was conducted on a total of 83 patients under the age of 10 who visited the Samsung Kids Pediatric Adolescent Clinic in Hwaseong, Gyeonggi Province, from September 2021 to March 2022, and met the diagnostic criteria of Hanifin and Rajka [26] for AD alongside 15 control subjects without any skin abnormalities confirmed through specialist diagnosis. Patients whose Objective SCORAD Index (OSI) scores were within the mild to moderate range, without exceeding the threshold for severe, were included to emphasize the need for preventive monitoring in patients with mild to moderate AD. Additionally, other skin diseases, chronic conditions, or systemic diseases that could not be included in the study due to accompanying factors were diagnosed and excluded by the dermatologist at Samsung Kids Pediatric Adolescent Clinic, apart from AD. This study obtained approval from the Institutional Review Board (IRB approval number EU21-068) of Eulji University prior to its execution. The purpose and methods of the research were explained to the research subjects and their guardians, and informed consent was obtained before the study commenced.

### 2.2. Study Design and Measurements

This study is a cross-sectional study with two groups of participants (AD patients and controls). For the initial assessment, to ensure objectivity in evaluating symptoms of AD, only the specialist's examination findings regarding the extent and intensity of the lesions were included. Subjective symptom assessments of itching and sleep disturbances were excluded. The OSI diagnostic method [24] was used for scoring, which quantifies the extent and intensity of the lesions. The total score ranged from 0 to 83, with scores below 15 indicating mild, scores between 15 and below 40 indicating moderate, and scores of 40 or above indicating severe AD [27]. Skin moisture measurement was conducted in a total of 15 selected areas known as common sites of AD occurrence: forehead, cheeks (left/right), neck (front/back), antecubital fossa (left/right), wrists (left/right), abdomen, back, popliteal fossa (left/right), and ankles (left/right) [28]. TEWL measurements were performed using the AF200 AquaFlux device (Biox Systems Ltd., England), and SCH measurements were conducted using the Corneometer CM 825 device (Courage-Khazaka, Köln, Germany). To ensure accuracy and reliability, both SCH and TEWL were measured three times at each site, with the average values being used for analysis. The measurements were conducted in a controlled environment with an average room temperature of 23°C ± 1°C and an average relative humidity of 50% to ensure consistent conditions in the same location.

### 2.3. Statistical Analysis

The collected data were statistically analyzed using SAS ver. 9.4 (SAS Institute Inc., Cary, USA). Descriptive statistics, such as mean and standard deviation, were calculated to examine the general characteristics of the subjects and the SCH and TEWL for each of the 15 body sites. Furthermore, the mean differences between the control group and the AD patient group for each body site were compared using Student's *t*-test to test for statistical significance, considering statistical methods to account for the disparity in sample sizes between the control and patient groups. The significance level for all tests was set at 0.05.

## 3. Results

The study included a total of 98 participants, with 15 in the control group and 83 in the patient group. The average age of the control group was 59 months, slightly higher than the patient group's average age of 50 months, but the difference was not statistically significant. The OSI for atopic patients was determined to be 15.4 ± 8. Based on the OSI scores, the AD patients fell within the range for moderate symptoms. SCH and TEWL measurements were taken for the 15 body sites of the study participants, and the average values for each body site were compared between the control and patient groups. The results showed that the patient group had lower SCH and higher TEWL values compared to the control group, but the differences were not statistically significant. The details are found in Table 1.

The basic statistics for the AD patient group are presented in Table 2. The atopic patients consisted of a total of 83 individuals, with 47 males (56.6%) and 36 females (43.4%). The average age of the patients was 54 months, with males being slightly older than females. The OSI scores were also slightly higher for males compared to females. Although males had lower SCH values and higher TEWL values compared to females, the difference between males and females in the AD group was not statistically significant.

The results of moisture retention (SCH) and moisture loss (TEWL) for the 15 body sites in the control group and AD patient group are shown in Table 3. Among the study participants, the head and neck region had the highest SCH compared to other body sites. The SCH values for the 15 body sites were slightly higher in the control group compared to the patient group, but the difference was not statistically significant. TEWL was highest at the wrists, and the differences in TEWL among the body sites were not significant. However, there were statistically significant differences in TEWL between the control and patient groups at the neck and antecubital fossa (inner elbow) body sites.

**Figure 1:**
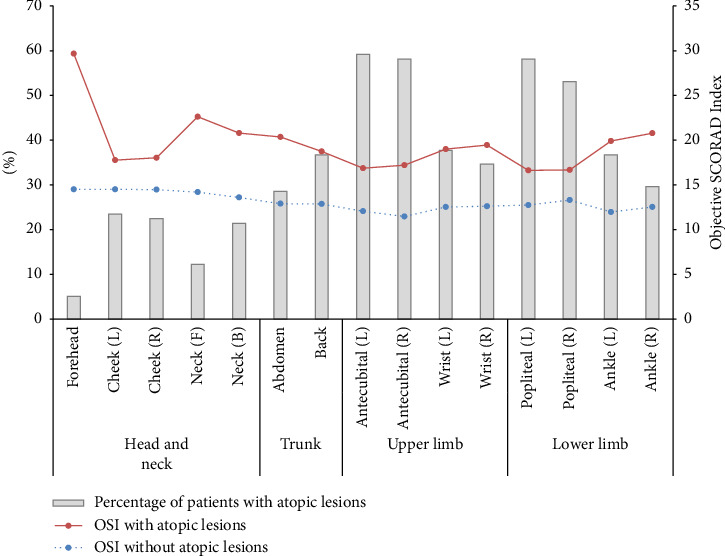
The percentage of patients with atopic lesions by 15 body parts and means of OSI. The bar graph shows the percentage of patients with atopic lesions, and the line graph represents the OCI values according to the presence or absence of atopic lesions.

Figure 1 illustrates the percentage of patients with atopic lesions at various body sites, along with their corresponding overall severity index values. Notably, 58.7% of the total atopic population exhibited frequent lesions in the antecubital fossa (inner elbow) and popliteal fossa (back of the knee) areas, while the proportion of patients with lesions in the head and neck regions was comparatively lower. When analyzing the OSI values based on the presence or absence of lesions at each body site, it was found that atopic patients with lesions consistently demonstrated higher OSI scores than those without lesions across all body sites. Interestingly, although the percentage of patients with lesions on the forehead was low, the mean OSI for those with forehead lesions was the highest, with a value of 29.7.

**Figure 2:**
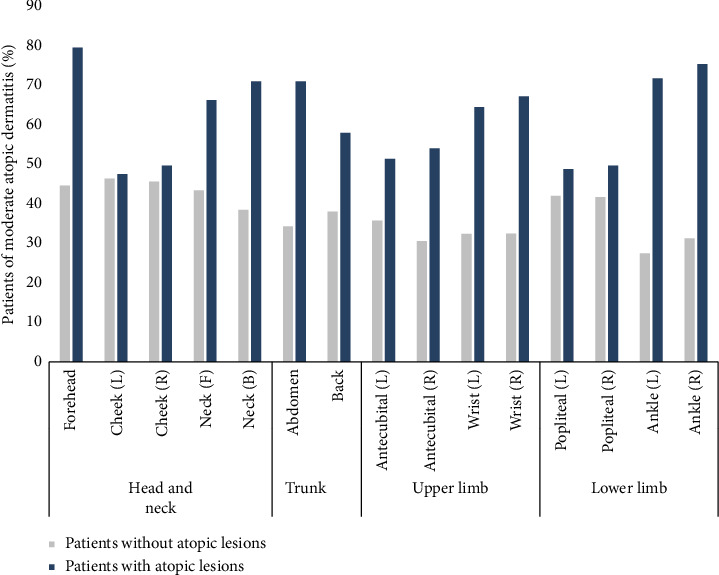
The percentage of patients with moderate atopic dermatitis according to atopic lesions by 15 body parts.

The percentage of moderate atopic patients based on the presence of atopic lesions for each body site is shown in Figure 2. It can be observed that the proportion of patients with moderate AD was higher in all body sites for the group of patients with lesions compared to the group without lesions. While the percentage of patients with moderate AD in the group without lesions was similar across body sites, the forehead and foot regions had a higher proportion of patients with moderate AD in the group with lesions.

**Figure 3:**
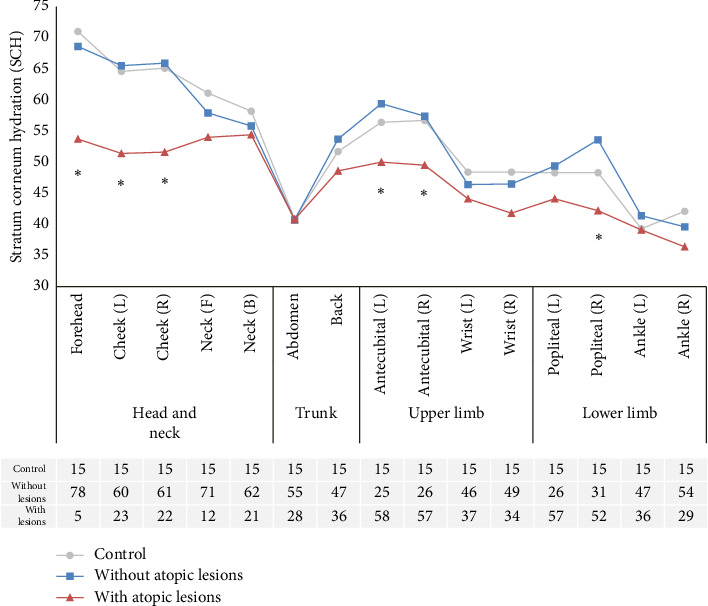
Stratum corneum hydration (SCH) values across 15 body parts in each group and corresponding sample sizes. Symbols (circles, squares, and triangles) stand for means. Significant difference between patients without atopic lesions and with atopic lesions (⁣^∗^*p* < 0.05).

The differences in SCH based on the presence of atopic lesions for each body site are shown in Figure 3. Overall, SCH was lower in areas other than the head and neck region, with the ankle region of the lower limb showing the lowest SCH. The abdomen region exhibited similar SCH levels between the control group and atopic patients, while in other body sites, atopic patients with lesions had the lowest SCH. Specifically, there were statistically significant differences in SCH between atopic patients with lesions and those without lesions in the forehead, cheek, antecubital fossa (inner elbow), and popliteal fossa (back of the knee) regions.

**Figure 4:**
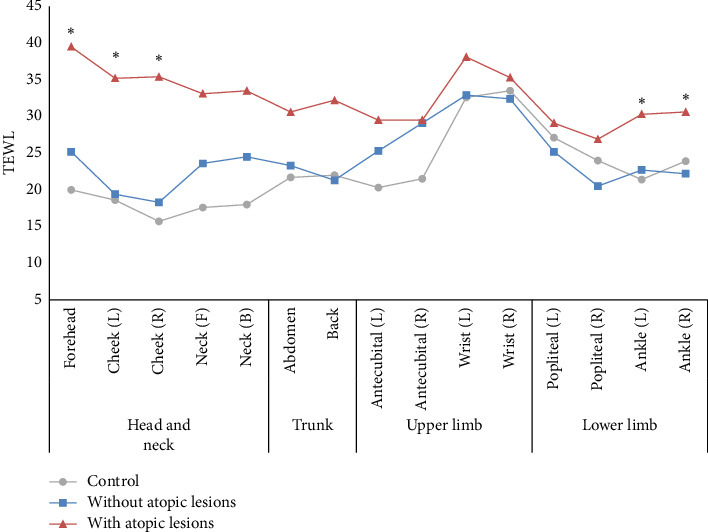
Transepidermal water loss (TEWL) values by 15 body parts in each group. Symbols (circles, squares, and triangles) stand for means. Significant difference between patients without atopic lesions and with atopic lesions (⁣^∗^*p* < 0.05).

The differences in TEWL based on the presence of atopic lesions for each body site are shown in Figure 4. There were no significant differences in TEWL based on atopic lesions in the face and popliteal fossa (back of the knee) regions. However, other body sites showed higher TEWL in patients with atopic lesions. Specifically, there were statistically significant differences in TEWL between atopic patients with lesions and those without lesions in the forehead, cheek, and ankle regions. The participants had the highest TEWL in the wrist area, and there was a noticeable decrease in TEWL from limb regions as the presence of atopic lesions increased in patients.

**Figure 5:**
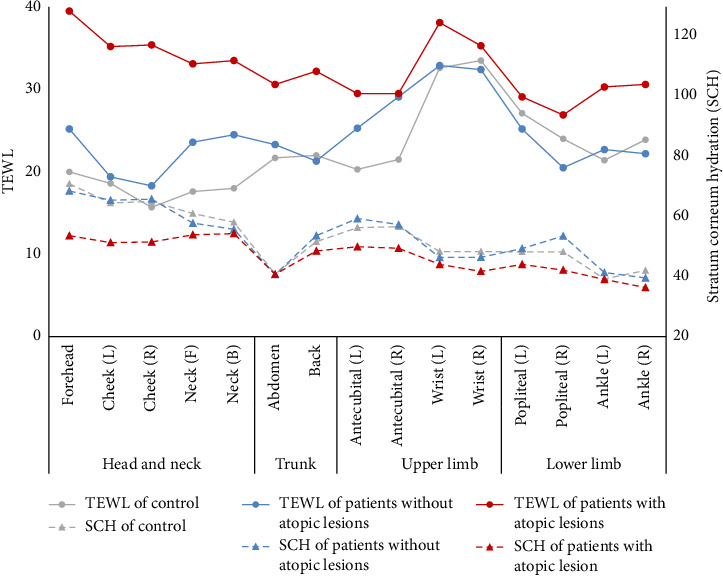
A graph demonstrating the relationship between stratum corneum hydration (SCH) and transepidermal water loss (TEWL) by 15 body parts in each group (circles for TEWL and triangles for SCH).

The relationship between SCH and TEWL by body site, as observed in different groups, is shown in Figure 5. There were not substantial differences in SCH among body sites, but significant differences were observed in TEWL. The differences in SCH and TEWL between body sites were more prominent in patients with atopic lesions. In particular, the head and neck region showed a significant difference. This study revealed that patients with atopic lesions exhibited lower SCH and higher TEWL in all body sites, and these differences were more pronounced compared to other groups.

## 4. Discussion

The evaluation of AD is essential for selecting treatment methods and improving their effectiveness [29]. Many studies are currently being conducted to develop various evaluation methods for AD, and the SCORAD index is the most widely used assessment method [30]. The SCORAD index evaluates both objective symptoms, such as the extent and severity of skin lesions, and subjective symptoms [27]. The OSI is a score that excludes subjective symptom assessment to enhance the objectivity of the evaluation [31]. Using OSI in the study participants, it was found that 53% (44 individuals) of AD patients were classified as mild, and 47% (39 individuals) were classified as moderate, with an average OSI score of 15.4 ± 8.1.

Some studies have reported that the SCORAD score reflects the subjective judgment of the observer and shows variability in the measurements taken by different observers [27, 32]. In light of this, recent research has focused on the applicability of skin barrier function, especially TEWL, as a predictive factor for the onset of conditions such as AD [33]. TEWL, which reflects the state of the skin's permeability barrier, along with SCH in the stratum corneum, is commonly used for evaluating skin barrier function [34]. In this study, we focused on the presence or absence of AD lesions in 15 specific body areas of the study participants using SCH and TEWL. The importance of site-specific SCH and TEWL measurements in pediatric AD patients was confirmed. Notably, areas with atopic lesions showed lower SCH and higher TEWL compared to nonlesional areas and the control group, representing a more advanced finding compared to previous patient-control studies.

Numerous studies on patients with AD have been conducted investigating the decrease in skin SCH [9] and the increase in TEWL [10, 11]. In this study, we observed low SCH and high TEWL in patients with AD, but the differences in SCH and TEWL between the control group and the patient group were not statistically significant. However, we were able to identify differences in SCH and TEWL among the 15 specific body areas based on the presence or absence of AD lesions. Body areas without AD lesions showed SCH and TEWL values that were not significantly different from those of the control group. On the other hand, significant differences were observed in SCH for the face, inner elbows, and forearms and in TEWL for the face and ankles in patients with AD. The face area in infants is known to be a representative site for the onset of AD in early childhood [35]. While there is a lack of previous studies specifically focusing on moisture measurements based on the presence or absence of AD lesions, it has been reported that impaired skin barrier function and increased moisture evaporation are key factors contributing to the development and exacerbation of AD, a condition with a heterogeneous and complex etiopathogenesis [13, 36]. Dryness, which serves as a key indicator of moisture loss, has been recognized as a major symptom of AD by the UK Working Group [37, 38]. This symptom plays a critical role in the diagnosis and management of the disease, as skin dryness is often linked to compromised skin barrier function. TEWL is commonly used to assess this barrier function, and several studies have demonstrated its efficacy as a scale to reflect the severity of AD symptoms [39]. In our study, we observed significant differences in both SCH and TEWL between patients with AD and control groups across most body areas. These findings underscore the importance of site-specific monitoring to better understand the progression of the disease and to tailor treatment approaches based on the unique characteristics of different body areas.

In this study, overall moisture levels were found to be highest in the face, neck, and trunk, with lower values observed in the limbs, except for the wrists. Notably, the forehead exhibited the highest moisture content in both the control group and the group without AD lesions, aligning with previous studies that report significantly higher moisture levels on the forehead compared to other facial regions, such as the cheeks. This difference is likely due to the denser concentration of sebaceous glands in the forehead, which enhances moisture retention. Studies utilizing modern skin hydration measurement techniques, such as the Corneometer, have consistently confirmed that the forehead retains more moisture compared to other parts of the face due to both anatomical and environmental factors, further supporting our findings [40, 41]. Among all the body areas, the wrists exhibited the lowest moisture levels and the highest rate of moisture loss. This finding is consistent with previous studies on exposed and nonexposed areas, which have demonstrated significantly higher TEWL values in exposed areas. The high moisture loss from the hands and wrists can be attributed to frequent contact with external factors that exacerbate skin conditions, such as handwashing, exposure to environmental elements, and irritants [42]. Additionally, this finding is particularly relevant considering the widespread use of hand sanitizers during the COVID-19 pandemic [43].

AD patients with lesions on the facial areas, particularly the forehead and cheeks, exhibited low SCH and high TEWL. Although the proportion of patients with lesions on the forehead and neck was low, the OSI had the highest average value of 30. This finding was especially pronounced in the group of patients with AD lesions, highlighting the relationship with the presence of these lesions on moisture levels. In the group with lesions, the forehead, cheeks, and ankles had a higher proportion of patients with moderate to severe AD compared to the group without lesions. This suggests that these areas, which have the highest moisture loss, can have a significant impact on the development of skin lesions. In this study, the participants were under the age of 10, and the highest proportion of patients with AD lesions was observed on the arms and legs (ranging from 50% to 60%), which are typical sites of AD occurrence after the age of 2. However, when considering the proportion of patients with moderate to severe AD based on lesion location, the forehead, ankles, and wrists showed a higher proportion of such patients. Considering that the typical sites of AD lesions and clinical symptoms can vary with age [44], and given that sites like the forehead, neck, ankles, and wrists become prevalent after the age of 12, it can be inferred that individuals under the age of 12 with AD lesions and low moisture levels in these areas are particularly vulnerable to AD. This highlights the need for preventive management of AD based on age and specific areas of the body.

According to a study [45], 93.7% of adult patients with AD had skin lesions on their faces, indicating a correlation between the presence of lesions and facial involvement. Although the proportion of patients with visible lesions was small, it was observed that they had the highest moisture loss. This suggests that if moisture loss persists, it may lead to the development of skin lesions. High moisture loss in the areas where lesions manifest after the age of 12 was confirmed, and it was also observed that areas with high moisture loss in children under the age of 10 were consistent with those seen in adult-onset AD. These findings suggest the need for site-specific moisture monitoring in patients with less severe conditions, as not all body sites are equally susceptible to onset of AD nor are they equally severe in symptom presentation. It also indicates that site-specific moisture monitoring can be utilized for predicting the onset and prognosis based on age as well as being used for preventive measures such as moisture management.

Although this study did not include evaluations based on age and severity across all age groups of AD patients, our larger cohort size and emphasis on atopic symptoms—alongside the conventional SCORAD scoring—added value by assessing 15 specific body areas, compared to previous research. The results strongly support the clinical benefit of incorporating body area-specific evaluations of moisture level into routine practice. Clinicians could implement this by systematically monitoring and recording symptom severity and moisture loss across different body areas, allowing for more precise, site-targeted interventions. For example, high-risk areas such as facial regions could be prioritized for intensified treatment or frequent reassessment. Tools such as wearable device or digital monitoring platforms could facilitate ongoing tracking of site-specific symptom progression, enabling real-time adjustments to treatment plans. By focusing on localized interventions—like targeted use of topical steroids, emollients, or localized phototherapy—clinicians can more effectively manage the disease's heterogeneity across different regions of the body [46]. Overall, these findings emphasize the importance of body-site specific monitoring and interventions in improving personalized care and long-term disease outcomes [47].

There are several limitations that should be acknowledged. First, the control group in this study was relatively small due to recruitment challenges. However, we employed statistical methods that account for differences in sample size to ensure valid patient-control comparisons. Despite the limited number of control participants, significant differences in SCH and TEWL were observed across specific body sites, depending on the presence or absence of skin lesions in atopic patients. Another limitation is the lack of continuous site-specific monitoring over time, which limited our ability to track changes in SCH and TEWL driven by external factors like environmental conditions and individual characteristics. These effects could not be fully captured in this cross-sectional design. Nonetheless, the site-specific data related to the presence or absence of skin lesions offer a useful reference point for future studies that incorporate continuous monitoring. Lastly, as this was an observational study, controlling for confounding variables was challenging. Still, we standardized SCH and TEWL measurements by ensuring they were taken from the same body sites under consistent environmental conditions (temperature and humidity) in AD patients. Despite these limitations, the study provides valuable insights. The area-specific assessments based on skin lesions offer foundational data for future research. Expanding the control group and incorporating more external factors in subsequent studies will enhance the robustness of the findings and lead to stronger conclusions. Considering variations in individual symptoms and seasonal influences will offer more precise insights for preventing symptom exacerbations and guiding therapeutic interventions for AD patients.

## 5. Conclusions

This study found significant differences in SCH and TEWL levels across various body areas in pediatric AD patients. While indices like SCORAD are valuable for assessing overall severity, they are less effective for targeted management based on specific body areas. Monitoring symptom progression according to the distribution of atopic lesions is crucial, as areas such as the face and ankles may have low moisture levels and high moisture loss, even in pediatric cases. Continuous site-specific monitoring is essential for preventing the spread of lesions and managing clinical symptoms more effectively.

## Figures and Tables

**Table 1:** Demographic data of control group and atopic dermatitis (AD).

Variable	Control	AD	*p* value
*N* (%)	15 (15.3%)	83 (84.7%)	NA
Age (month), mean ± SD	59.2 ± 22.5	50.9 ± 30.0	0.313
OSI, mean ± SD	NA	15.4 ± 8.1	NA
SCH (a.u.), mean ± SD	53.4 ± 8.5	50.9 ± 8.5	0.297
TEWL (g/m^2^/h), mean ± SD	25.7 ± 18.3	28.5 ± 14.8	0.428

Abbreviations: a.u., arbitrary unit; NA, not applicable; OSI, Objective SCORAD Index; SCH, stratum corneum hydration; TEWL, transepidermal water loss.

**Table 2:** Demographic data of atopic dermatitis (AD) patients.

Variable	Male	Female	*p* value
*N* (%)	47 (56.6%)	36 (43.4%)	NA
Age (month), mean ± SD	54.3 ± 33.0	46.5 ± 25.4	0.244
OSI, mean ± SD	16.0 ± 8.8	14.7 ± 7.2	0.479
CorM, mean ± SD	49.4 ± 8.8	52.8 ± 7.7	0.076
TEWL, mean ± SD	31.1 ± 17.8	25.1 ± 8.9	0.051

*Note:* Significantly different by *t*-test.

Abbreviations: a.u., arbitrary unit; NA, not applicable; OSI: Objective SCORAD Index; SCH, stratum corneum hydration; TEWL, transepidermal water loss.

**Table 3:** Skin hydration and TEWL by 15 body parts in control and atopic dermatitis (AD) patients (mean ± SD).

		SCH		TEWL	
		Control	AD	Control	AD
Head and neck	Forehead	71.0 ± 7.9	67.7 ± 14.1	20.0 ± 14.3	26.2 ± 15.1
	Cheek (L)	64.6 ± 17.3	61.5 ± 19.4	18.6 ± 7.9	24.1 ± 16.9
	Cheek (R)	65.1 ± 13.6	62 ± 18.1	15.7 ± 8.1	23.2 ± 15.5
	Neck (F)	61.1 ± 14.0	57.3 ± 15.4	17.6 ± 8.1	25.1 ± 18.4⁣^∗^
	Neck (B)	58.2 ± 13.7	55.9 ± 12.3	18.0 ± 5.3	28.4 ± 21.3⁣^∗∗^

Trunk	Abdomen	40.9 ± 15.0	40.7 ± 13.5	21.7 ± 14.0	25.9 ± 20.0
	Back	51.7 ± 18.1	51.5 ± 13.8	22.0 ± 26.9	26.5 ± 24.1

Upper limb	Antecubital (L)	56.4 ± 10.7	52.8 ± 14.5	20.3 ± 9.5	28.5 ± 23.8⁣^∗^
	Antecubital (R)	56.7 ± 14.3	51.9 ± 13.5	21.5 ± 10.8	29.4 ± 21.7⁣^∗^
	Wrist (L)	48.4 ± 12.1	45.4 ± 11.7	32.6 ± 27.2	35.4 ± 20.9
	Wrist (R)	48.4 ± 12.9	44.5 ± 13.2	33.5 ± 17.2	32.6 ± 17.1

Lower limb	Popliteal (L)	48.3 ± 16.4	45.8 ± 14.7	27.1 ± 16.8	28.1 ± 19.7
	Popliteal (R)	48.3 ± 15.5	46.3 ± 14.6	24.0 ± 10.1	25.1 ± 15.2
	Ankle (L)	39.3 ± 11.2	40.4 ± 11.8	21.4 ± 10.5	26.1 ± 13.1
	Ankle (R)	42.1 ± 17.4	38.4 ± 11.3	26.9 ± 16.0	25.6 ± 13.8

*Note:* Significantly different by *t*-test.

⁣^∗^*p* < 0.05.

⁣^∗∗^*p* < 0.01.

## Data Availability

The informed consent given by this study's participants does not cover data sharing or posting in publicly accessible databases. However, the data will be available upon request by means of a project agreement from the authors. Requests should be sent to haha0694@skuniv.ac.kr and are subject to approval by all named authors participating in this study.
